# Identification of single nucleotide polymorphisms from the transcriptome of an organism with a whole genome duplication

**DOI:** 10.1186/1471-2105-14-325

**Published:** 2013-11-16

**Authors:** Kris A Christensen, Joseph P Brunelli, Matthew J Lambert, Jenefer DeKoning, Ruth B Phillips, Gary H Thorgaard

**Affiliations:** 1 School of Molecular Biosciences, Washington State University, Pullman WA 99164-4660, USA; 2 School of Biological Sciences, Washington State University, Pullman WA 99164-4236, USA; 3 School of Biological Sciences, Washington State University, Vancouver, 14204 NE Salmon Creek Ave, Vancouver WA 98686-9600, USA; 4 Center for Reproductive Biology, Washington State University, Pullman WA 99164-7520, USA

**Keywords:** SNP, Polyploid, Rainbow trout, Genome duplication

## Abstract

**Background:**

The common ancestor of salmonid fishes, including rainbow trout (*Oncorhynchus mykiss*), experienced a whole genome duplication between 20 and 100 million years ago, and many of the duplicated genes have been retained in the trout genome. This retention complicates efforts to detect allelic variation in salmonid fishes. Specifically, single nucleotide polymorphism (SNP) detection is problematic because nucleotide variation can be found between the duplicate copies (paralogs) of a gene as well as between alleles.

**Results:**

We present a method of differentiating between allelic and paralogous (gene copy) sequence variants, allowing identification of SNPs in organisms with multiple copies of a gene or set of genes. The basic strategy is to: 1) identify windows of unique cDNA sequences with homology to each other, 2) compare these unique cDNAs if they are not shared between individuals (i.e. the cDNA is homozygous in one individual and homozygous for another cDNA in the other individual), and 3) give a “SNP score” value between zero and one to each candidate sequence variant based on six criteria. Using this strategy we were able to detect about seven thousand potential SNPs from the transcriptomes of several clonal lines of rainbow trout. When directly compared to a pre-validated set of SNPs in polyploid wheat, we were also able to estimate the false-positive rate of this strategy as 0 to 28% depending on parameters used.

**Conclusions:**

This strategy has an advantage over traditional techniques of SNP identification because another dimension of sequencing information is utilized. This method is especially well suited for identifying SNPs in polyploids, both outbred and inbred, but would tend to be conservative for diploid organisms.

## Background

Whole genome duplications followed by differentiation of many gene duplicates in the ancestors of rainbow trout and other salmonid fishes [1,2] have created one of the most complex animal genomes [3-5]. Historical polyploid events have also been important in the ancestry of all vertebrates [6,7], and are of broad significance in plant evolution [8,9]. Genome duplications increase the complexity of genomic studies [10] and result in a need to distinguish between paralogs [11,12]. Population and quantitative genetics fundamentally rely on the accurate identification of the alleles of a gene or locus [13].

Identification of alleles is complicated when paralogous genes or sequences are easily mistaken for alleles [14]. Recent analytical methods have increasingly turned to single nucleotide polymorphisms (SNPs) as the preferred genetic marker to distinguish between alleles because they are ubiquitous and can be readily scored [15]. SNPs are easily mistaken for paralogous sequence variants because many paralogs are similar in sequence and may contain common variants that were present before genome duplication.

**Figure 1:**
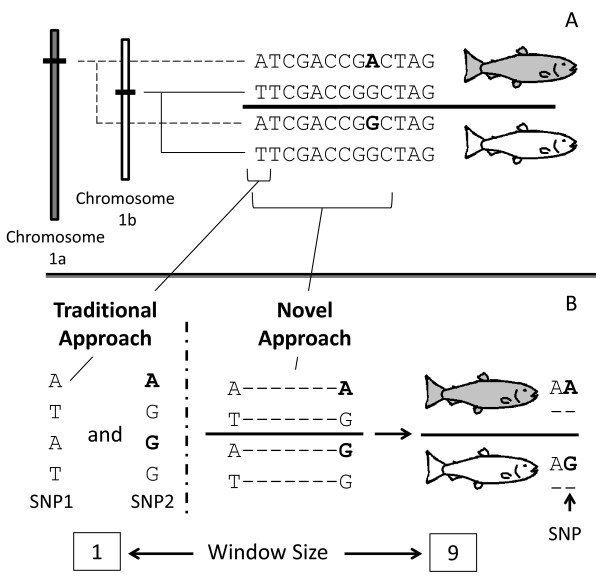
**A comparison between traditional approaches and our strategy of SNP identification.** Panel **A** shows two homeologs (duplicated chromosomes) and the sequence of both alleles from each chromosome. The fish are homozygous and only have one allele from each homeolog. This means that SNPs can only be found by comparing sequences between the two homozygous fish. Any variation found within a fish is a paralogous sequence variant (i.e. the first nucleotide in the gene sequence). Panel **B** illustrates the differences between traditional approaches and ours. The traditional approach would scan every nucleotide position and identify any variant as a possible SNP. This leads to a 50% error rate for the illustrated case (SNP1 is actually a paralogous sequence variant). Our approach uses windows of sequence to identify potential SNPs. Variant windows, in this case 9 nucleotides, are compared between individuals, with windows found in both individuals not considered for SNP identification.

Traditional SNP calling programs use sequence alignments of cDNA or genomic sequence and identify any variant position as a possible SNP [16]. If a paralog is included in the alignment, traditional strategies will incorrectly identify paralogous sequence variants as possible SNPs (Figure 1). Traditional approaches of transcriptome data analysis for SNP identification have been utilized for both diploid and polyploid organisms [17,18].

In the diploid carrot, 18% of the potential SNPs were found to be false-positives (excluding technical failures) using a traditional method of analyzing transcriptomic data [17], and in some fish species the false-positive rate was very high (e.g. catfish 41.4%) [18]. In fishes with polyploid ancestries, the false-positive rate was 19.4% for whitefish [19], and 15.9% for chum salmon [20].

Two main non-traditional approaches have been used to distinguish SNPs from paralogous sequence variants. The most common strategy has been to remove all polymorphisms associated with genes that have paralogs [21-25]. Such filtering drastically reduces the total number of genes in which polymorphisms can be detected in species with recent polyploidy ancestries and may still lead to a relatively high false-positive rate. A filtering strategy was used for a diploid coral transcriptomic dataset, which resulted in a 30% false-positive rate [26] and a 12% false-positive rate was found for ryegrass using a similar strategy [27]. In polyploid alfalfa, a window approach was used to eliminate SNPs if the potential alleles differed by 10% or more in the window surrounding the SNP [28]. This resulted in a false-positive rate of 37.5%, a very positive outcome considering that the alfalfa was both polyploid and had heterozygous genotypes. In salmonids, filtering resulted in a false-positive rate of 68.6% [29].

Other researchers have proposed strategies to detect polymorphisms without filtering paralogs [30-32]. These methods allow identification of differences between alleles in genes with paralogs, but alleles will still be indistinguishable from paralogs in future studies if the sequence is not reported with the SNP. Knowledge of a SNP at a given location is not fully informative as paralogs may share common sequence with an allele.

The SNP calling method described here involves the analysis of windows of sequence (similar to [28]) and only comparing homozygous alleles differing between individuals (Figure 1). We utilized homozygous clonal lines of rainbow trout because they ensure homozygous alleles at every locus and any difference within an individual likely represents a paralogous sequence variant. This strategy may also be used for SNP identification in outbred organisms, but only SNPs from homozygous loci will be identified.

## Results

### cDNA output, SNP calling, and paralog counting

Embryo and head kidney cDNA were sequenced for five homozygous clonal lines of rainbow trout. The average number of reads per clonal line was 114,096 (SD = 67 k) for the embryo and 105,464 (SD = 71 k) for the head kidney (Table 1). cDNA reads were aligned to the unique rainbow trout UniGene database (build #34) with Megablast version 2.2.21 [33] and with Novoalign version 3.00.05 [34] to compare these two alignment programs.

**Table 1:** Counts of cDNA reads from embryo and head kidney tissues from five rainbow trout lines

	**Embryo**	**Head kidney**	**Both**
Arlee (YY)	96077	95196	191273
Clearwater (YY)	231981	108170	340151
Skamania (XX-male)	64002	126150	190152
Swanson (YY)	90235	12	90247
Whale Rock (XX)	88186	197793	285979
All	570481	527321	1097802

**Figure 2:**
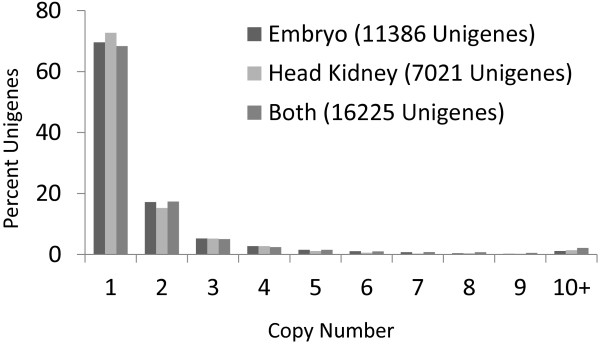
**UniGene copy number distribution in rainbow trout embryo and head kidney tissues.** The graph shows the percent of UniGenes that have evidence for specific copy numbers, from a single copy gene to ten or more copies. The alignment program used was Megablast with default settings.

For each UniGene sequence, the aligned cDNA reads were used to estimate the number of paralogs and identify potential SNPs for both tissues (Table 2). Figure 2 shows the distribution of different categories of gene duplication for the different tissues and the combined result. SNPs were identified in 4,533 of the UniGene reference sequences and 7,412 (Additional file 1) total SNPs were found when both tissues and all the clonal lines were combined (SNP score > 0.25, indels not included, Megablast alignments). For Novoalign alignments, SNPs were found in 2,979 references, with 5,052 total SNPs identified. The number of SNPs detected between clonal lines of rainbow trout ranged from 792 to 3451 (Table 3).

**Table 2:** Gene duplication counts and SNPs for rainbow trout embryo and head kidney tissues

	**Single copy UniGenes**	**Multiple copy UniGenes**	**SNPs**
**Embryo**	5170 (7923)	754 (3458)	3337 (5034)
**Head kidney**	2566 (5101)	399 (1906)	1376 (2284)
**Both**	7289 (11100)	1234 (5113)	5052 (7412)

The number of single and multiple copy UniGenes was estimated using the reads from all clones from the different tissues (The highest copy number from all individual clonal lines was used as the combined estimate for a UniGene). UniGenes without sufficient evidence of copy number are not represented. The SNP counts were found by comparing all clones against each other at the same time. These SNPs have a SNP score of 0.25 or greater, meaning that all of them have more than a single read representing each allele. The first set of numbers are from Novoalign alignments and the second set are from Megablast (in parentheses).

**Table 3:** A comparison of SNPs between clonal rainbow trout lines for the combined tissues

	**Arlee**	**Clearwater**	**Skamania**	**Swanson**	**Whale rock**
Arlee	-	2292	1460	1180	1628
Clearwater	(3300)	-	1425	1283	2412
Skamania	(2109)	(2156)	-	792	1486
Swanson	(1722)	(1846)	(1197)	-	1182
Whale Rock	(2359)	(3451)	(2191)	(1716)	-

The number of SNPs with SNP scores of 0.25 or greater using sequences from both embryo and head kidney tissues between the clonal lines of rainbow trout. The alignments, used to identify these SNPs, were produced using Novoalign with default settings (top) and Megablast (bottom in parentheses).

The number of SNPs between lines appears to be correlated with number of reads, with the lines having higher read counts also having more SNPs (Tables 2 and 3). A value of 0.25 for the SNP score was used as a threshold for reporting based on Table 4 (discussed below), and because an allele only represented by one read would score below this value (discussed in Methods section). Removing these alleles should remove most sequencing errors. The embryo and head kidney tissues had the same gene counts for roughly half of the overlapping UniGenes (2326 of 4782) when using Megablast alignments and 72% (1340 of 1858) using Novoalign alignments.

**Table 4:** SNP validation comparison using a transcriptome dataset from polyploid wheat

	**Original study**	**Window 80,**	**Window 80,**	**Window 40,**	**Window 10,**
		**SNP score > 0**	**SNP score > 0.25**	**SNP score > 0**	**SNP score > 0**
Validated SNPs	64	40 (37)	28 (22)	35 (34)	18 (23)
False-positives	61	3 (6)	1 (0)	2 (1)	3 (8)
False-positive rate	49%	7% (14%)	3% (0%)	5% (3%)	14% (26%)

The number of SNPs from the original study and from different settings using our strategy. There are multiple numbers per parameter explored. These reflect different alignment programs used under these settings. The first set of numbers were from Novoalign alignments, and the second were from Megablast (in parentheses).

**Figure 3:**
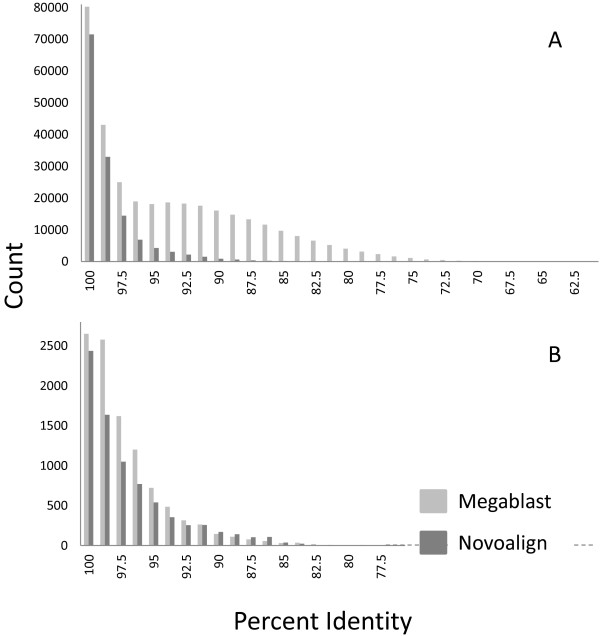
**Alignment comparison.** Panel **A** is a histogram of sequence similarity of alignments for the Clearwater clonal line. Alignments were produced by either Megablast or Novoalign and for every 80 nucleotide window the alignments were binned into their respective similarity category based on the number of mismatches in the window. The second peak of alignments around 92.5% with Megablast likely represents a set of genes duplicated during a whole genome duplication. Novoalign does not show this pattern because Novoalign uses a more stringent alignment algorithm. Panel **B** was produced in the same way, but it represents the combination of two polyploid wheat lines for 125 UniGenes used in SNP validation. The second peak for this panel is around 98.75% indicating a much more recent whole genome duplication. Both alignment programs have similar results with wheat because the paralogs have high sequence similarity.

The different SNP numbers and percent gene duplication (~32% Megablast and ~14% Novoalign), identified while using different alignment programs resulted from how alignments were produced. Novoalign uses a global alignment algorithm and produced fewer alignments between diverged paralogs (Figure 3). The Novoalign program produces relatively few alignments with lower sequence identity compared to the Megablast program. The Megablast result is similar to an all-against-all EST comparison from Atlantic salmon with greater divergence between ancestral genomes in the rainbow trout [35].

In a general sense, having few low sequence identity alignments indicates that the Novoalign program is more accurate because diverged paralogs are not aligning to the reference sequences. However, our strategy requires the alignment of paralogs so we can distinguish between these sequences and identify SNPs in paralogs that are not represented in the reference sequences.

### SNP validation

**Figure 4:**
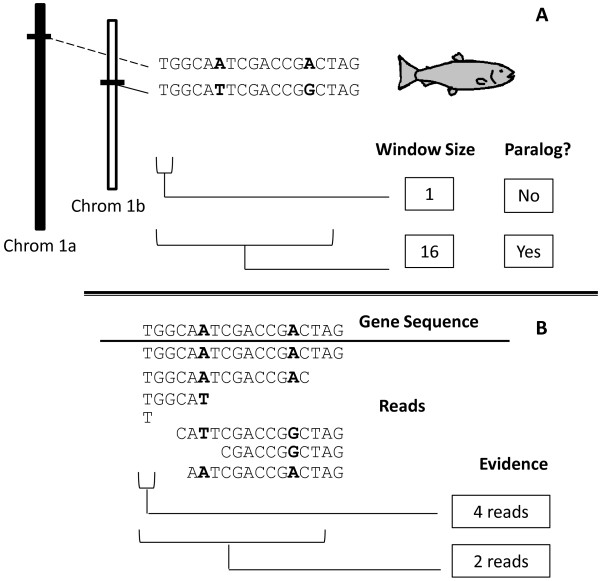
**Effect of window size on distinguishing paralogs and the amount of useable data.** Panel **A** shows two homeologs and the sequence of paralogs from a homozygous fish. Paralogous sequence variants are in bold text. Two windows are represented (1 and 16), only the 16 nucleotide window can differentiate between the paralogs. Panel **B** shows a multiple sequence alignment of the expressed cDNA reads aligned to the gene sequence from Chrom 1a. Depending on the alignment criteria and the sequence similarity, the two different paralogs will both align to the sequence from Chrom 1a. The number of useable sequences depends heavily on the window size. The larger the window size, the less likely that a read will span the window length, but the more likely the paralogs will be distinguishable.

We downloaded cDNA and reference sequences used by another research group to identify SNPs in polyploid wheat [36]. We applied our strategy for SNP discovery to these previously validated UniGenes, allowing for direct comparison of validation rates (Table 4). Variation in window size can produce differing results (Figure 4). Larger window sizes produced fewer false-positives (Table 4), and adopting a SNP score threshold of 0.25 eliminated all false-positives. The false-positive rate varied by alignment program, but in all instances the false-positive rate was lower using the strategy described here than was observed in the original study (Table 4).

There was little variance in SNP validation when comparing different alignment programs. During SNP identification in rainbow trout, nearly twice as many SNPs were identified when Megablast was used. In wheat, SNP identification is virtually the same between alignment programs. It appears from the 125 gene dataset that the genome duplication event was much more recent in wheat than in rainbow trout. For this reason both alignment programs were able to align the highly similar paralogs with similar performance (Figure 3). It is believed that there was an allopolyploid event in tetraploid wheat less than 0.5 MYA [37], which corresponds to highly similar paralogous sequence seen in this dataset.

### Sequencing validation

For SNP validation ten sets of primers were also created to amplify genomic DNA of the different trout lines of ten predicted SNPs in sequences downloaded from NCBI. Five of these pairs amplified well and were polymorphic at the predicted site when sequenced. The low amplification rate is attributed to the lack of information related to repetitive sequences and introns surrounding the window in which a SNP was predicted (technical errors). The five that amplified well had scores of: 0.35, 0.35, 0.41, 0.42, and 0.8. The others had scores of: 0.04, 0.37, 0.42, 0.57, and 0.76.

## Discussion

Traditional methods of SNP identification are effective for standard diploid organisms, but are not appropriate for organisms that have had a genome duplication. Other methods have been employed to overcome traditional limitations [30-32], but they may be of limited use for future studies if they cannot effectively integrate the occurrence of paralogs into SNP identification. Our strategy incorporates such information during SNP calling and may be used for both inbred lines and outbred populations.

The importance of alignment program selection increases as paralogs diverge. Early after a genome duplication, it is likely that most alignment programs could effectively align paralogous sequences together. As mutations alter the paralog sequences, high stringency alignment programs will no longer align paralogs together. SNP discovery in diploids benefits from accurate alignments, but information about the paralogs is much more valuable in polyploids than highly precise alignments.

Dependence on a nucleotide window is both a weakness and strength of this strategy. While modifications may be made to increase or decrease the window size, doing so may also influence the results. For example, a decreased window size may limit the ability to distinguish similar paralogs (Figure 4). Alternatively, an increased window size would allow the identification of all non-identical paralogs, but would decrease the likelihood that a cDNA sequence would span the window completely and would decrease the amount of usable data. Traditional strategies have an effective window size of one nucleotide and for this reason are unable to differentiate between paralogs.

One distinctive component of our strategy is the stringent use of only homozygous alleles in SNP identification. This eliminates many false-positive SNPs that are the result of paralogous sequence variants. At the same time, fewer SNPs will be found because heterozygous alleles are eliminated during SNP identification. To maximize SNP identification, it may be more appropriate to use a low stringency method and use allele frequency as a filter instead of the homozygous selection method described here.

A complementary application of this method might be in the identification of gene families or paralogs. Our method tries to identify paralogs from short cDNA sequences in order to score SNPs, but the estimation may be useful in other ways. For example, the described strategy may be used as an initial method for identifying genes that may be duplicated. Identification and enumeration of paralogs would benefit gene expression, phylogenetic, and gene ontology studies.

## Conclusions

The SNP validation evidence suggests that our method offers an advantage over existing strategies of SNP identification for polyploid organisms. This strategy might also be used for analyzing data derived from diploid organisms, but would likely be overly conservative for SNP identification in those organisms, resulting in identification of fewer SNPs.

## Methods

### mRNA isolation, cDNA synthesis, and 454 pyrosequencing

Total RNA was isolated from tissues of five different clonal lines of doubled haploid rainbow trout. Arlee, Clearwater, and Swanson are YY male lines [38], Skamania is apparently an XX male line with a female-to-male sex reversal mutation (Paul Wheeler, personal communitication), and the Whale Rock line is an XX female line [30]. RNA was extracted from approximately 30 eyed-stage embryos (~15 days post fertilization at 11°C) from each clonal line. The yolk sacs were removed from the embryos in a chilled 0.9% saline solution before RNA extraction. Head kidney RNA was also isolated from an ~ one-year-old fish for all five clonal lines.

Tissues were suspended in TRIzol (Invitrogen), flash frozen in liquid nitrogen, and stored at −80°C until RNA extraction. Tissue samples were homogenized using a glass Dounce tissue grinder as recommended in the TRIzol RNA extraction protocol [39]. After TRIzol phase separation, two additional rounds of RNA extraction were performed using a four to one ratio of acidic phenol (pH ~4.5):chloroform, followed by a final chloroform extraction. Total RNA was precipitated with isopropyl alcohol, washed with 70% ethanol, and suspended in water. cDNA was synthesized from each total RNA source using the SMARTer™ PCR cDNA synthesis protocol (Clontech) and was amplified with Advantage PCR according to the manufacturer’s instructions. Amplified cDNAs were purified by phenol-chloroform extraction [40] and precipitated with an equal volume of isopropyl alcohol, washed with 70% ethanol, and suspended in water.

The samples were then sent to the Genomics Core at Washington State University where the cDNA quality was assessed using an Agilent DNA 1000 kit (Agilent Technologies). Nebulization of the cDNA followed, using the 454 Rapid DNA Library protocol (Roche). Each cDNA sample was then barcoded and each cDNA library was quantified using a PerkinElmer VICTOR^tm^ X. Libraries were titrated by small volume emulsion PCR and then pooled into two libraries for large volume emulsion PCR. Finally, both libraries were sequenced using a Roche 454 FLX Titanium Genome Sequencer on a single picotiter plate.

### Sequence alignment

Barcoded cDNA sequences were separated based on their clonal line of origin and tissue using Roche’s SFF Tools [41] and modified to conform to standard FASTA using custom scripts. They were deposited to the NCBI’s sequence read archive (study accession: SRP028233). The cDNA dataset for each clonal line and each tissue was then aligned to the rainbow trout unique UniGene database build #34 [42] using default settings of standalone Megablast (version 2.2.21). Novoalign (version 3.00.05) was used to create the same alignments with default settings.

The default setting for the number of database sequence alignments to report for Megablast is 250, and the number of high-scoring segment pairs (HSPs) to save per database sequence is unlimited. These settings may produce pseudo-homologous alignments, but hypothetically may be beneficial in SNP discovery because they help us identify regions of sequence where we have little confidence. Sequence regions with low complexity (if not filtered) or high genomic copy number will produce many false alignments, and these regions will appear to have many extra copies when compared to the full reference UniGene sequence. A method for handling these circumstances is described later.

Novoalign uses a global alignment algorithm, while Megablast uses a local alignment algorithm. These two types of alignment strategies were used to determine how much SNP discovery and gene copy estimation relied on the type of alignment program used. It was expected that global alignment algorithms would be better suited for aligning similar sequence, and as a result fewer gene copies would be estimated using this strategy because dissimilar paralogs would not align.

The unique rainbow trout UniGene database contains 142,505 sequences and was generated by clustering publicly available sequences of high similarity together [42]. From each cluster the longest sequence was taken as a representative for that cluster [42]. If copies of a gene are represented in the databases and are of sufficient similarity, they would be clustered together and only one representative would remain. This is important for estimating the number of genes that have been copied in the reference database. If there were genes with extra copies represented twice, then the estimate would be biased.

### Paralog counting

Homozygous organisms are expected to have a single copy of every allele, if there is evidence for more than one allele, it likely indicates the presence of a paralog with similar sequence (Figure 4). To estimate the number of copies a gene has, each UniGene reference sequence alignment was examined for unique cDNA types using custom Perl scripts (Additional file 2). Estimating gene copy number was done to improve SNP identification, as described below.

First, a reference sequence was split into overlapping windows, and for each of these windows all unique cDNA types with at least two copies were counted. The database sequence was split into windows because often cDNA sequence reads are short and in order to identify variant cDNA types, they need to cover the same distance. This means that only those portions of cDNA that completely span a window will be used in paralog counting (and in SNP calling later). A window size of 80 nucleotides was used for all rainbow trout analyses. cDNAs that have a sequence similarity of ~99% would on average have one polymorphism in each 80 nucleotide window. cDNA types were identified by looking for differences between the cDNA read and the reference sequence in the alignments. The differences were recorded relative to the reference sequence, making divergent cDNA types comparable. Each cDNA type had a unique pattern of differences from the reference sequence.

Next, multiple windows, representing at least half of the reference sequence, with the same copy number were used as a threshold to reduce the misleading effect of repetitive motifs on the paralog count estimation. If the highest count of cDNA types did not cover 50% of the database sequence (e.g. one window had 10 different cDNA types and all the other windows had 2 different cDNA types), then the count of cDNA types in those windows was reduced by one and the new number of cDNA types was checked for the threshold. This was done until the count was found in 50 percent of the windows or until the count reached zero.

Fifty percent was used instead of 100% because it is expected that even with perfect coverage, there will be some windows with fewer cDNA types than the true paralog number because paralogs may have complete sequence similarity in those windows.

### SNP calling

For each of the UniGene sequence windows (described in the previous section), the cDNA types within those windows were compared if all the clonal lines were homozygous for either cDNA type (Figure 1). cDNA types found within a homozygous individual are not alleles and were not compared. The comparisons were then given a SNP score based on several criteria: sequence similarity, number of nearly identical paralogs, number of cDNA types in a window compared to the full UniGene sequence, coverage, even representation of alleles, and the phred quality score at the SNP position. These scores are not probabilities; they reflect the level of confidence that a variant represents a SNP (and not a paralog or one of several classes of errors) based on the previously mentioned criteria. The SNP score representational formulation is: SNP Score = (sequence similarity^10^)^Nearly identical paralogs^ * paralog information * sequencing error * unequal representation and coverage * sequencing quality score. Each of the elements from this equation are discussed below. The score will range from zero to one.

#### Sequence similarity

The initial SNP score was determined by taking the sequence identity and raising it to the tenth power (Additional file 3). For example, if two cDNA types had 0.99 sequence similarity, they would be given a score of 0.904. A variable threshold was then used to eliminate unlikely comparisons (e.g. < ~0.99 similarity). The sequence similarity was raised to the tenth power because comparisons with modest sequence similarity (e.g. 0.95, score = 0.599) are assumed to be much more likely due to paralog differences than to allelic differences. The window size will greatly affect this score (Additional file 3).

#### Nearly identical paralogs

If a cDNA type was compared to multiple cDNA types, all comparisons with that cDNA type had their scores lowered. Multiple comparisons may be expected if there is expression of a gene and its paralog in one of the individuals being compared, but only expression of the gene in the other individual. The SNP scores were raised to the power of (a + 1)*10, where “a” is the number of comparisons with the cDNA type above 1 (Additional file 3). This expression was chosen to effectively eliminate known paralog differences from further consideration if a SNP score of 0.25 was used as a threshold (Additional file 3). It is possible that one of the comparisons would be between alleles, but identifying which would require more information.

#### Paralog information

To further distinguish between SNP and paralog differences, any window with a low or high paralog count relative to the rest of the UniGene reference sequence (estimate was found in the previous section), was scored lower (Additional file 3). If a particular window had more cDNA types than the paralog count estimated over the whole sequence, then it may contain a repetitive motif and any SNPs identified in these windows may be less reliable because of the sheer number of comparisons being made. If there were fewer cDNA types in a window, it may mean there is less information or that it was a conserved sequence and any SNPs found in this window may be shared among paralogs (and thus are not the locus-specific SNPs we are seeking). The modification was different depending on the type of comparison, as explained below.

#### Insufficient data for a window

If the cDNA count for a window was estimated to be zero, then the SNP score was multiplied by the following expression: 1/c (Additional file 3). The ‘c’ was the estimated paralog count for the whole database sequence. We expect to see windows with estimated cDNA counts of zero when there is insufficient data and therefore we are less confident about any SNPs found in these windows. As the number of estimated paralogs increases, our confidence for which paralog is represented in the window, with insufficient data, decreases (e.g. 1/1, 1/2, 1/3 using the equation above).

#### Insufficient data for the entire database sequence

If there were few representations of cDNA types along half of the database sequence (i.e. the paralog count was zero), then the SNP scores were multiplied by (0.5/b)^2^ (Additional file 3). The ‘b’ in the equation was the number of cDNA types for the window with the SNP. If the length of a database sequence has poor sequence coverage, but a single window contains ample evidence, we assumed that the window with strong evidence is likely a repetitive motif. The more cDNA types found in one of these windows, the less confidence we had for any SNPs in that window (e.g. 1 cDNA type = > 0.25, 2 = > 0.06, 3 = > 0.03 using the equation above).

#### Repetitive gene motifs

If a window had more cDNA types than that estimated for the whole sequence (and the paralog count was not zero), the score was multiplied by (c/b)^^2^, or (b/c)^^2^ if the window had fewer cDNA types (Additional file 3). If a window contains more cDNA types than the whole database sequence, then it may mean that this window contains repetitive motifs or conserved sequence. Both interpretations decrease the confidence that a SNP found in a window with high numbers of cDNA types is real (e.g. 2 copies in window: 1 copy for whole gene = > 0.7, 3:1 = > 0.6 using the equation above). Low coverage and increased sequence similarity between paralogs may cause windows to have fewer cDNA types than the rest of the database sequence. This decreases the likelihood that a SNP is real in windows with fewer cDNA types.

#### Sequencing error

To distinguish SNPs from sequencing errors, the score was reduced by 75 percent if there was only a single representation (i.e. one cDNA read) of an allele (Additional file 3). If the scores were not reduced, the majority of the high scoring SNPs (> 0.25) were those with a single representative (data not shown). While these SNPs may be valid, they lack enough evidence to be considered high-confidence.

#### Unequal representation and coverage

The SNP score was also lowered if there was an overabundance of one allele compared to the other. It is assumed that alleles will have roughly similar expression profiles, and many sequencing errors will be unequally represented. The SNP score was multiplied by (1 + (x + y)/2)/2 * (4 + x/y)/5, where ‘x’ is the number of the low count allele and ‘y’ is the number of the high count alleles (Additional file 3). This equation was used because it incorporates coverage and even representation, with deviations from equal representation lowering the score. At a lower coverage, unequal representation causes a greater deviation than it would at higher coverage (Additional file 3). We expect most sequencing errors to be at lower coverage.

#### Sequencing quality score

Every SNP score was modified by multiplying the score by the average of the sequencing quality scores (of all cDNA reads) assigned to the nucleotides at the SNP’s location divided by the best score possible. If this score is near one, then it will not appreciably modify the SNP score, but if the sequencing quality scores are low, the SNP score will be proportionally lowered. If the sequencing score is low, then it is likely that the proposed SNP is actually a sequencing error and not real. Insertions and deletions were not considered candidate SNPs.

### Alignment program comparison

Novoalign and Megablast use different alignment algorithms. To test how this affects how many alignments are produced for our dataset, we examined the combined tissues of the Clearwater clonal line. After the reference sequences were broken into windows as described above, we used custom Perl scripts to count the alignments in each window. Every alignment was binned into an appropriate sequence identity category based on the number of mismatches in that window. The same was done for the wheat dataset, except the data from the two wheat lines were combined after binning.

### SNP validation

We downloaded cDNA sequences (NCBI sequence read archive files ERR045179 and ERR045180) and reference sequences (*Triticum aestivum* UniGene build #62 ) used by another research group to identify SNPs in polyploid wheat [36]. The strategy they used accounted for the polyploid nature of wheat, but they had an effective window size of one nucleotide. We then used custom Perl scripts to retrieve the sequences used in their SNP validation analysis (both the correctly identified SNPs and those that were monomorphic) from the downloaded UniGene database.

These 125 sequences were used as a reference set. The cDNA sequence sets were transformed into fastq files using the NCBI’s sratoolkit version 2.1.9 [43] and paired-end reads were split into separate files at the same time. Fastq files were converted to fasta files with quality scores using custom Perl scripts for use with Megablast. Megablast and Novoalign were then used to align the cDNA sequences against the reference set.

Megablast uses a local alignment algorithm, Smith-Waterman, while Novoalign uses a global alignment algorithm, Needleman-Wunsch. Local alignment algorithms are generally used for dissimilar sequence alignment, while global alignment algorithms are more for similar sequence alignment. Default settings were used for Novoalign and Megablast. The different alignment program outputs were in SAM format or BLAST format. Custom scripts described above were used to identify potential SNPs. Perl scripts were used to compare the identified SNPs to the validated SNPs.

### Sequencing validation

Ten primers were designed [44] surrounding ten proposed rainbow trout SNPs with a range of scores (0.04 - 0.8 using Megablast alignments) to see if the genomic sequence corresponded to cDNA sequence. These proposed SNPs were selected to minimize insertions/deletions in between any known paralogs because they could have interfered with sequencing. The primers were found in ten reference sequences downloaded from the NCBI (BT045031, BT045191, FR677582, AF042218, AB208024, AJ627208, AY029216, AY593999, AY370888, AY065837). The primers were checked against a preliminary draft of the rainbow trout genome to make sure they did not align to splice sites (obtained from Michael Miller, University of California, Davis).

### Availability of supporting data

cDNA sequences were deposited in the sequence read archive of the NCBI (http://www.ncbi.nlm.nih.gov/sra/?term=SRP028233). Perl scripts and instructions on how they may be used are available in Additional file 2.

## Competing interests

The authors declare that they have no competing interests.

## Authors’ contributions

KAC wrote the Perl scripts, conducted the bioinformatics analyses, and drafted the manuscript. JPB prepared cDNA from clonal lines and helped draft the manuscript. MJL and JD sequenced candidate SNPs. GHT and RBP conceived of and initiated the study and helped draft the manuscript. All authors read and approved the final manuscript.

## Supplementary Material

Additional file 1 **SNPs Found Between Five Rainbow Trout Lines in Rainbow Trout.** SNPs found between five rainbow trout clonal lines in UniGene build #34 reference sequences. This file contains the sequence and genotype information for the SNPs found using the Megablast alignment program. This file can be opened with a text editor or with a spreadsheet program, information is separated by tabs. Click here for file

Additional file 2 **Perl Scripts and Instructions.** This file must be decompressed before it is opened. It contains a folder with the various Perl scripts used for SNP discovery and instructions on how they may be used. The instructions may be opened with a text editor (e.g. notepad ++). Click here for file

Additional file 3 **SNP Score Generation.** Panel A illustrates the initial SNP score based on sequence similarity for three different nucleotide window sizes. In all cases the initial SNP score decreases quickly as the number of mismatches increases. Panel B shows how three different initial scores are modified if evidence for a nearly identical paralog is found. In all cases the score is lowered below a SNP score of 0.25. Panel C represents SNP scores after paralog information is analyzed. The X axis represents the number of estimated paralogs for a given window, while the different categories represent different numbers of estimated paralogs for the entire reference sequence. Panel D characterizes how the SNP score is modified by coverage and differential representation of the alleles. The X axis represents the coverage of allele 1 and the different categories represent the coverage of allele 2. Click here for file
