# Supplementary material for: Identification of single nucleotide polymorphisms from the transcriptome of an organism with a whole genome duplication
Source: BMC Bioinformatics. 2013 Nov 16;14:325. doi: 10.1186/1471-2105-14-325 (PMC3840595; doi:10.1186/1471-2105-14-325)
Supplement: Additional file 3 — SNP Score Generation. Panel A illustrates the initial SNP score based on sequence similarity for three different nucleotide window sizes. In all cases the initial SNP score decreases quickly as the number of mismatches increases. Panel B shows how three different initial scores are modified if evidence for a nearly identical paralog is found. In all cases the score is lowered below a SNP score of 0.25. Panel C represents SNP scores after paralog information is analyzed. The X axis represents the number of estimated paralogs for a given window, while the different categories represent different numbers of estimated paralogs for the entire reference sequence. Panel D characterizes how the SNP score is modified by coverage and differential representation of the alleles. The X axis represents the coverage of allele 1 and the different categories represent the coverage of allele 2. [file 1471-2105-14-325-S3.pptx]

## Slide 1
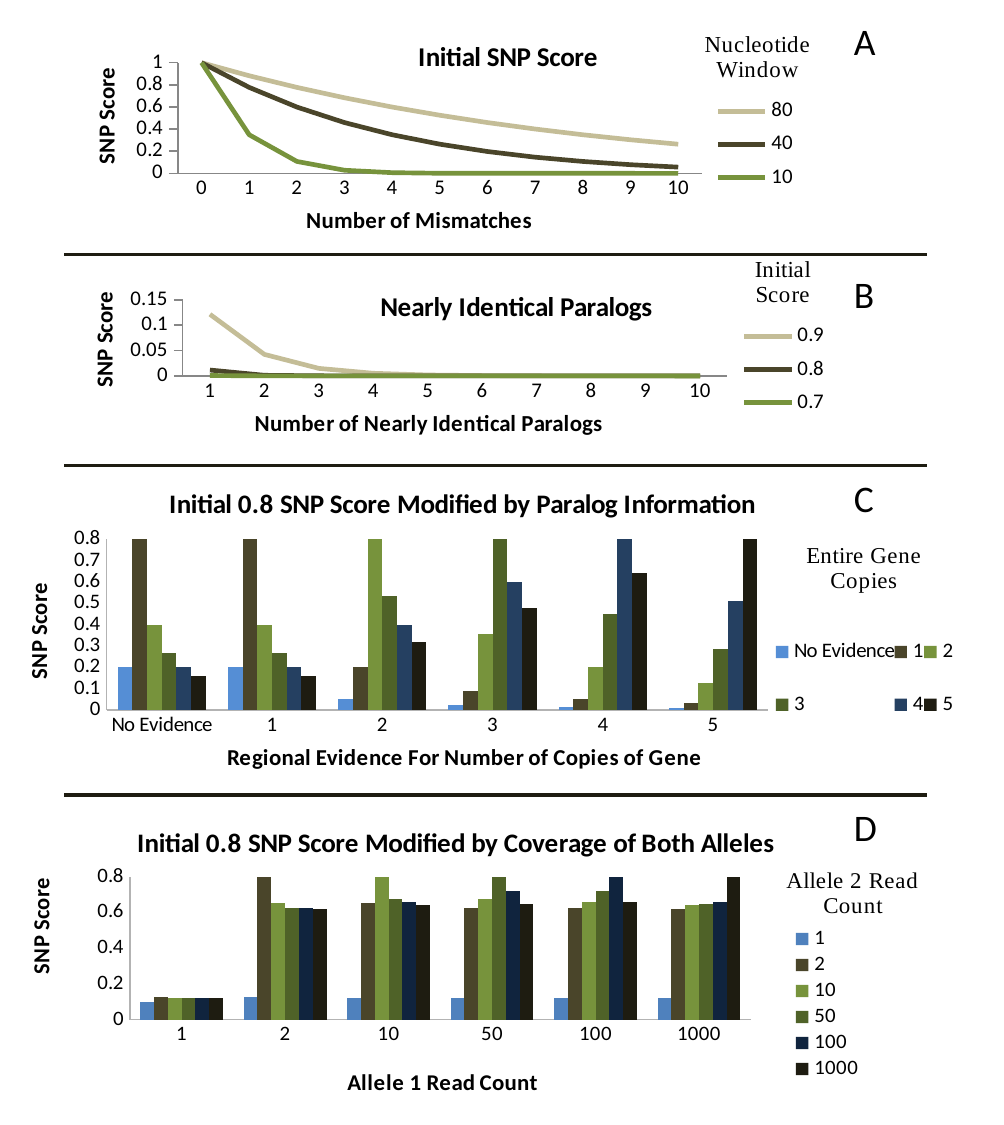

A
### Chart: Initial SNP Score
| Category | 80 | 40 | 10 |
|---|---|---|---|
| 0 | 1.0 | 1.0 | 1.0 |
| 1 | 0.88180192584422 | 0.776329620856438 | 0.3486784401 |
| 2 | 0.776329620856438 | 0.598736939238379 | 0.1073741824 |
| 3 | 0.682350292111393 | 0.458582341424737 | 0.0282475249 |
| 4 | 0.598736939238379 | 0.3486784401 | 0.0060466176 |
| 5 | 0.524460475048727 | 0.263075576163828 | 0.0009765625 |
| 6 | 0.458582341424737 | 0.196874404340723 | 0.0001048576 |
| 7 | 0.40024759524535 | 0.146062754179425 | 5.9049e-06 |
| 8 | 0.3486784401 | 0.1073741824 | 1.024e-07 |
| 9 | 0.303168180492695 | 0.0781658446293641 | 1e-10 |
| 10 | 0.263075576163828 | 0.0563135147094727 | 0.0 |
### Chart: Nearly Identical Paralogs
| Category | 0.9 | 0.8 | 0.7 |
|---|---|---|---|
| 1 | 0.121576654590569 | 0.0115292150460685 | 0.000797922662976122 |
| 2 | 0.0423911582752162 | 0.00123794003928538 | 2.25393402906923e-05 |
| 3 | 0.0147808829414346 | 0.000132922799578492 | 6.36680576090905e-07 |
| 4 | 0.00515377520732012 | 1.42724769270596e-05 | 1.79846504264742e-08 |
| 5 | 0.00179701029991443 | 1.53249554086589e-06 | 5.08021860739626e-10 |
| 6 | 0.000626578748217798 | 1.64550455732121e-07 | 1.43503601609869e-11 |
| 7 | 0.000218474500528393 | 1.76684706477839e-08 | 4.05362155971447e-13 |
| 8 | 7.61773480458666e-05 | 1.8971375900642e-09 | 1.14504775943211e-14 |
| 9 | 2.65613988875875e-05 | 2.0370359763345e-10 | 3.23447650962479e-16 |
| 10 | 9.2613871309979e-06 | 2.18725072478303e-11 | 9.13659557440914e-18 |B
C
### Chart: Initial 0.8 SNP Score Modified by Paralog Information
| Category | No Evidence | 1 | 2 | 3 | 4 | 5 |
|---|---|---|---|---|---|---|
| No Evidence | 0.2 | 0.8 | 0.4 | 0.266666666666667 | 0.2 | 0.16 |
| 1 | 0.2 | 0.8 | 0.4 | 0.266666666666667 | 0.2 | 0.16 |
| 2 | 0.05 | 0.2 | 0.8 | 0.533333333333333 | 0.4 | 0.32 |
| 3 | 0.0222222222222222 | 0.0888888888888889 | 0.355555555555556 | 0.8 | 0.6 | 0.48 |
| 4 | 0.0125 | 0.05 | 0.2 | 0.45 | 0.8 | 0.64 |
| 5 | 0.008 | 0.032 | 0.128 | 0.288 | 0.512 | 0.8 |D
### Chart: Initial 0.8 SNP Score Modified by Coverage of Both Alleles
| Category | 1 | 2 | 10 | 50 | 100 | 1000 |
|---|---|---|---|---|---|---|
| 1 | 0.1 | 0.129375 | 0.122959 | 0.1205996784 | 0.1202999599 | 0.12002999995999 |
| 2 | 0.129375 | 0.8 | 0.650832 | 0.6261987072 | 0.6230998392 | 0.62030999983992 |
| 10 | 0.122959 | 0.650832 | 0.8 | 0.671830656 | 0.655835836 | 0.6414395998396 |
| 50 | 0.1205996784 | 0.6261987072 | 0.671830656 | 0.8 | 0.71999838 | 0.647998703838 |
| 100 | 0.1202999599 | 0.6230998392 | 0.655835836 | 0.71999838 | 0.8 | 0.655999835836 |
| 1000 | 0.12002999995999 | 0.62030999983992 | 0.6414395998396 | 0.647998703838 | 0.655999835836 | 0.8 |
